# Influence of the propagation strategy for obtaining robust *Saccharomyces cerevisiae* cells that efficiently co-ferment xylose and glucose in lignocellulosic hydrolysates

**DOI:** 10.1111/1751-7915.12280

**Published:** 2015-05-18

**Authors:** Elia Tomás-Pejó, Lisbeth Olsson

**Affiliations:** 1 Department of Biology and Biological Engineering, Industrial Biotechnology, Chalmers University of TechnologySE-412 96, Gothenburg, Sweden; 2 Unit of Biotechnological Processes for Energy Production, Instituto Madrileño De Estudios Avanzados (IMDEA) Energy28935, Móstoles (Madrid), Spain

## Abstract

Development of xylose-fermenting yeast strains that are tolerant to the inhibitors present in lignocellulosic hydrolysates is crucial to achieve efficient bioethanol production processes. In this study, the importance of the propagation strategy for obtaining robust cells was studied. Addition of hydrolysate during propagation of the cells adapted them to the inhibitors, resulting in more tolerant cells with shorter lag phases and higher specific growth rates in minimal medium containing acetic acid and vanillin than unadapted cells. Addition of hydrolysate during propagation also resulted in cells with better fermentation capabilities. Cells propagated without hydrolysate were unable to consume xylose in wheat straw hydrolysate fermentations, whereas 40.3% and 97.7% of the xylose was consumed when 12% and 23% (v/v) hydrolysate, respectively, was added during propagation. Quantitative polymerase chain reaction revealed changes in gene expression, depending on the concentration of hydrolysate added during propagation. This study highlights the importance of using an appropriate propagation strategy for the optimum performance of yeast in fermentation of lignocellulosic hydrolysates.

## Introduction

The yeast *Saccharomyces cerevisiae* has been used for centuries by many cultures for baking and producing alcoholic beverages. Furthermore, during the past few decades, this yeast has been exploited to produce bioenergy – in particular, bioethanol.

The conversion of abundant lignocellulosic biomass into bioethanol is a sustainable alternative to the present industrial production of bioethanol, which uses starch and sucrose-derived feedstocks as raw materials. Fermentation of lignocellulose-derived materials is, however, very challenging for *S. cerevisiae* because apart from sugars, inhibitory compounds derived from cellulose, hemicellulose and lignin degradation during pretreatment of the biomass are also present in the resulting hydrolysate (Alvira *et al*., 2010). The inhibitory compounds prevent the growth of yeast and affect fermentation performance. Furthermore, wild-type *S. cerevisiae* is unable to ferment xylose, which can constitute up to 40% of the lignocellulosic material. Only enteric bacteria and some fungi and yeasts are able to ferment xylose but with low yield (Tomás-Pejó, 2011). Some ethanologenic bacteria like *Escherichia coli* have shown promising alternatives for industrial exploitation (Okuda *et al*., 2007). However, wild-type *E. coli*, for example, shows low ethanol yield because it converts sugar more efficiently to organic acids and several approaches have been performed with the aim of redirecting glycolytic fluxes to ethanol (Tao *et al*., 2001).

In order to upgrade the implementation of lignocellulosic bioethanol production to industrial scale, it is imperative to develop robust xylose-fermenting strains that work efficiently under the prevailing conditions. One commonly used strategy to enable wild-type *S. cerevisiae* to consume xylose is to introduce the *XYL1* and *XYL2* genes from *Scheffersomyces stipitis*. These genes encode xylose reductase (XR) and xylitol dehydrogenase (XDH) respectively. However, because of the different preferences of XR and XDH for co-factors, xylitol formation is one of the main drawbacks of using this strategy – leading to a lower yield of ethanol from xylose than from hexoses.

Because it has been shown that xylose fermentation capacity is more affected by inhibitors than glucose fermentation (Martín *et al*., 2007; Ask *et al*., 2013), the development of yeast strains that are tolerant to inhibitors is even more important when xylose-fermenting traits are introduced. During the last years, several studies involving metabolic and evolutionary engineering have been performed to obtain xylose-fermenting *S. cerevisiae* strains that work efficiently in lignocellulosic hydrolysates (Koppram *et al*., 2012; Kim *et al*., 2013; Gonçalves *et al*., 2014). In this context, it has been shown that evolutionary engineering (i.e. long-term adaptation) of xylose-fermenting strains to lignocellulosic hydrolysate not only leads to better results in terms of tolerance to inhibitors, but also to an increase in xylose fermentation capacity and ethanol yield compared with non-adapted cells (Martín *et al*., 2007; Tomás-Pejó *et al*., 2010).

Propagation of the seed culture is an important step in every fermentation process that demands efficient production of yeast cells with high fermentative efficiency. Exposing the cells to the inhibitory hydrolysate during the propagation step would allow short-term adaptation of the yeast cells to the inhibitors during pre-inoculum growth (Koppram *et al*., 2013). In this work, the effect of short-term adaptation during propagation on cell metabolism and gene expression is studied for the first time.

Molecular adaptation to different growth conditions during the propagation step is, however, poorly understood. In the last years, some studies have been carried out to analyse the complexity of the yeast biomass production process for wine strains (Gómez-Pastor *et al*., 2011), but little is known about how the propagation conditions influence the capacity of the cells to produce bioethanol from lignocellulosic materials.

In the present study, we assessed the importance of the propagation strategy for obtaining robust strains that efficiently co-ferment glucose and xylose in lignocellulosic hydrolysates. The engineered industrial strain *S. cerevisiae* KE6-12 harbouring the xylose genes (XR and XDH) from *S. stipitis* and overexpressing the endogenous xylulokinase was used as the fermenting microorganism. This strain was previously developed to grow well on lignocellulosic hydrolysates (Albers, E., Halpin, R. and Olsson, L. *et al*., unpublished) and has been successfully used in bioethanol production processes from lignocellulose (Moreno *et al*., 2013; Tomás-Pejó *et al*., 2014). However, in those cases, xylose consumption was not higher than 75% in 120–144 h, highlighting the importance of the propagation strategy to improve xylose consumption.

We studied the effect of the different propagation strategies on the specific growth rate in minimal medium with two inhibitory compounds (vanillin and acetic acid). Vanillin is a phenolic aldehyde compound that has been shown to be one of the most potent inhibitors as it inhibits fermentation at very low concentration (Klinke *et al*., 2004; Nguyen *et al*., 2014). On the other hand, acetic acid is a carboxylic acid commonly found in high concentration in lignocellulosic hydrolysates that contributes to cell arrest and reduction of ethanol productivities (Mira *et al*., 2010).

The performance of cells propagated under different conditions was compared during ethanol production processes from wheat straw hydrolysate. We also determined the influence that the addition of hydrolysate during propagation has on the expression of key genes known to be related to the stress response and tolerance to inhibitors.

## Results and discussion

### Propagation strategy

Depending on the propagation strategy, different concentrations of wheat straw hydrolysate were added during the cell propagation step. In the base case, no hydrolysate was used, but only a defined medium. The wheat straw hydrolysate was recovered after filtering the whole pretreated slurry. The latter had 20.4% (w/w) total solids and was obtained after pretreating the wheat straw at 190°C for 15 min in a steam explosion plant, by SEKAB in Örnsköldsvik, Sweden.

Wheat straw hydrolysate had the following composition: 14.5 g l^−1^ glucose, 32.6 g l^−1^ xylose, 3.5 g l^−1^ arabinose, 1.8 g l^−1^ mannose, 8.5 g l^−1^ acetic acid, 1.6 g l^−1^ formic acid, 1.3 g l^−1^ 5-hydroxymethyl furfural (HMF), 7.7 g l^−1^ furfural and 0.05 g l^−1^ vanillin.

For the propagation, pre-inocula were grown at 30°C in 250 ml Erlenmeyer flasks shaken at 150 r.p.m. containing 50 ml of Delft medium as follows: 10 g l^−1^ glucose, 20 g l^−1^ xylose, 7.5 g l^−1^ (NH_4_)_2_SO_4_, 3.5 g l^−1^ KH_2_PO_4_, 0.75 g l^−1^ MgSO_4_·7H_2_O, 2 ml l^−1^ trace metal solution and 1 ml l^−1^ vitamin solution (Verduyn *et al*., 1990). Wheat straw hydrolysate, filtered and adjusted to pH 6, was added at the mid-exponential phase of pre-inoculum growth (OD_600 nm_ ≈ 2.5) when the cells were starting to grow on xylose. According to the propagation strategy, wheat straw hydrolysate was added to reach a final concentration of 12% or 23% (v/v) in the pre-inoculum medium. The sugar content in 12% (v/v) hydrolysate was increased to have the same sugar content as present in 23% (v/v) hydrolysate. Furthermore, minimal medium only (designated 0% hydrolysate) with sugar content corresponding to that of 23% (v/v) was also used for comparative purposes.

After addition of hydrolysate, the pre-inoculum culture was grown for another 8 h. The specific growth rates for the propagation cultures were similar under the three conditions investigated.

### Cell growth in the presence of acetic acid and vanillin

In order to check whether the propagation strategy had an effect on the tolerance to acetic acid and vanillin, the growth of cells propagated under different conditions was tested in 96 micro-well equipment Bioscreen C MBR (Growth Curves, Helsinki, Finland). The medium used was Delft minimal medium with 10 g l^−1^ glucose, 20 g l^−1^ xylose, 1 g l^−1^ vanillin, and 5 or 10 g l^−1^ acetic acid. The concentration of acetic acid was in the range of what would be predictable in steam-exploded wheat straw hydrolysates, but the concentration of vanillin (1 g l^−1^) was 10 times higher than what would be expected, in order to make the effect more evident (Tomás-Pejó *et al*., 2010).

**Figure 1:**
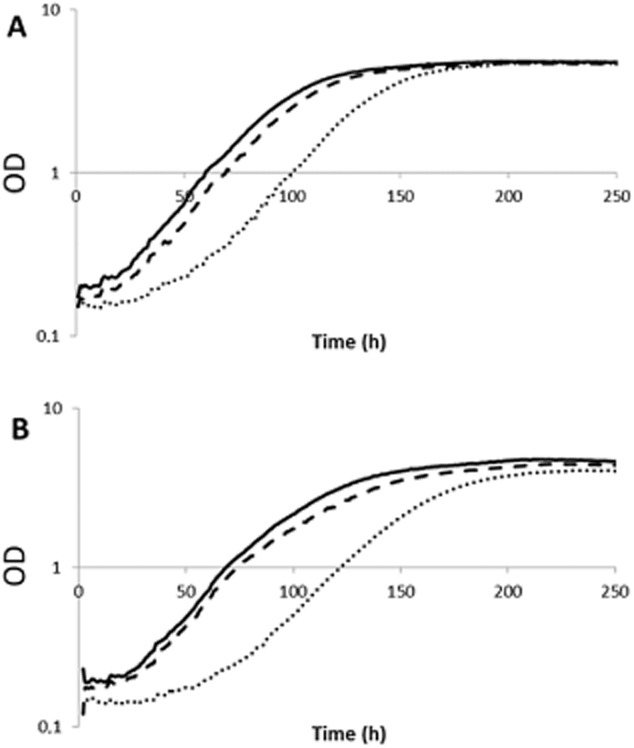
Growth in minimal medium with 10 g l^−1^ glucose and 20 g l^−1^ xylose; growth curves of cells propagated with (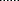) 0% (v/v) hydrolysate, (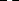) 12% (v/v) hydrolysate or (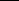) 23% (v/v) hydrolysate in the presence of (A) 5 g l^−1^ acetic acid and 1 g l^−1^ vanillin or (B) 10 g l^−1^ acetic acid and 1 g l^−1^ vanillin. Ten growth curves were analysed for each condition for 3 days at 30°C in a working volume of 145 μl. Initial OD580 nm was 0.1. OD580 nm values were taken every 15 min using a wide-band filter with λ = 420–580 nm.

It was clear that cells propagated with different hydrolysate concentrations (0, 12 or 23% (v/v)) had different growth curves in the presence of acetic acid and vanillin (Fig. 1).

In minimal medium with 5 g l^−1^ acetic acid and 1 g l^−1^ vanillin (Fig. 1A), the specific growth rates were 0.02 h^−1^, 0.08 h^−1^ and 0.10 h^−1^ with cells propagated at 0%, 12% and 23% (v/v) hydrolysate respectively. On the other hand, the specific growth rates in the presence of 10 g l^−1^ acetic acid and 1 g l^−1^ vanillin were 0.009 h^−1^, 0.07 h^−1^ and 0.08 h^−1^ with cells obtained at 0%, 12% and 23% (v/v) hydrolysate respectively (Fig. 1B). Thus, as expected, the specific growth rates in the presence of 10 g l^−1^ acetic acid and 1 g ^l1^ vanillin were lower than in the presence of 5 g l^−1^ acetic acid and 1 g l^−1^ vanillin. As predicted, the specific growth rate was always higher when the cells were propagated in the presence of 23% (v/v) hydrolysate than with 12% and 0% hydrolysate. Furthermore, the lag phase was longer in cells propagated without any hydrolysate (0% v/v) than with 12% and 23% hydrolysate, irrespective of the acetic acid concentration.

Mira and colleagues (2010) showed that after subjecting *S. cerevisiae* cells to a certain level of acetic acid, growth arrest occurred but cell growth was resumed after a lag phase. However, when these pre-adapted cells were used to re-inoculate medium under the same conditions with same acetic acid concentration, no delay in cell growth was observed what would suggest that some changes at genomic level occur during the adaptation. In a similar way, our results also showed that cells pre-grown in the presence of acetic acid – as was the case for cells propagated with 12% and 23% hydrolysate – were more able to tolerate acetic acid (Fig. 1).

### Fermentation experiments

To study the differences in ethanol production and xylose fermentation capacity according to the propagation strategy, cells propagated with different concentration of hydrolysate were used to ferment wheat straw hydrolysate diluted to 50% (v/v). This concentration was fixed according to previous experiments to have an inhibitory hydrolysate without compromising the fermentability of the medium.

After propagation, cells were harvested by centrifugation at 5000 r.p.m. for 5 min at room temperature, and the cell pellet was weighed and diluted with sterile water to obtain the desired inoculum size for the fermentation experiments (1.5 g dry weight of cells l^−1^).

**Figure 2:**
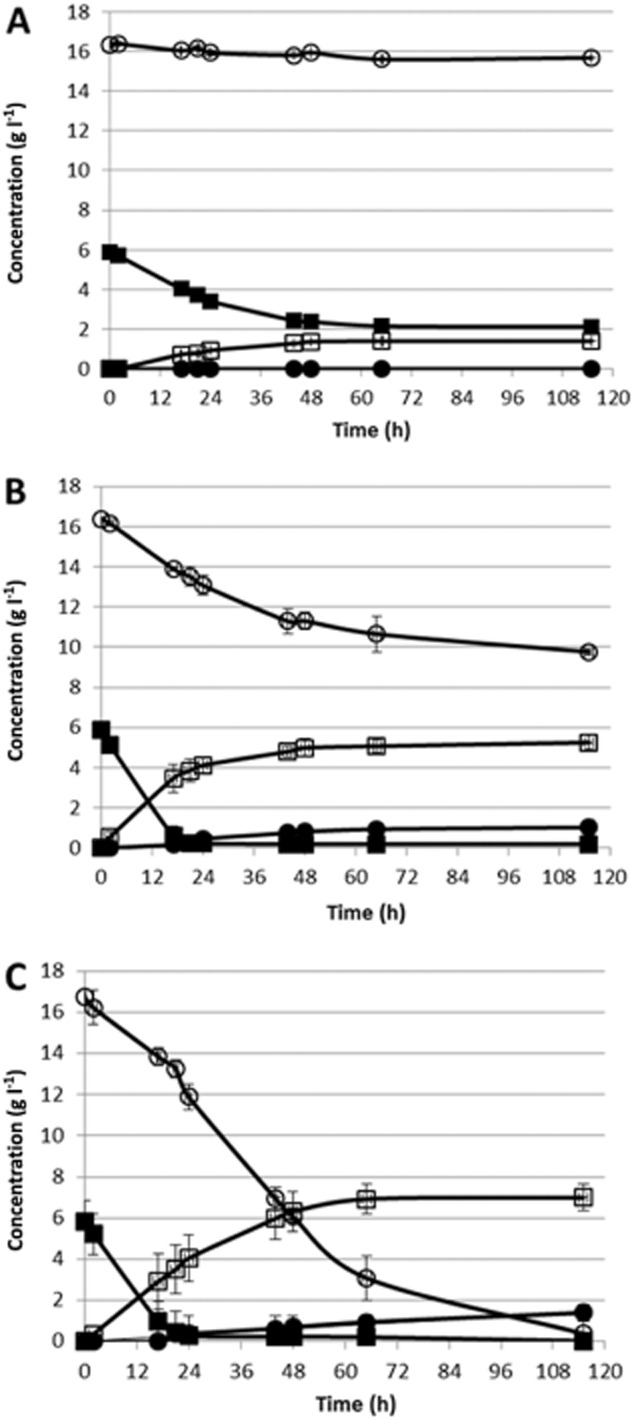
(○) Xylose, (●) xylitol, (■) glucose and (□) ethanol levels in fermentation of 50% (v/v) diluted wheat straw hydrolysate. Experiments were performed for 115 h at 30°C and pH 5.5, in 150 ml semi-anaerobic shake flasks using glycerol traps to allow CO_2_ outflow and no inflow of oxygen. Di-ammonium phosphate at 0.5 g l^−1^ was used as nitrogen source. The inoculum size was fixed at 1.5 g l^−1^ dry weight. Hydrolysate added during the propagation step: (A) 0% (v/v), (B) 12% (v/v) and (C) 23% (v/v).

When 0% (v/v) hydrolysate was added during the propagation, no xylose was consumed in the following fermentation step (Fig. 2A). On the other hand, 40% and 98% of the xylose was consumed when 12% and 23% (v/v) hydrolysate, respectively, was added during the propagation (Fig. 2B and C). Also, the glucose fermentation capacity was affected by the addition of hydrolysate during the propagation because in the case where there was no hydrolysate addition, the wheat hydrolysate was very inhibitory to the yeast and only 4 g l^−1^ of glucose was consumed in 115 h. Glucose, however, was depleted in less than 24 h when 12% or 23% (v/v) hydrolysate was used during the propagation. The improved xylose and glucose co-fermentation capacity in hydrolysate-propagated cells was translated into an increase in ethanol yield from total sugars, from 0.24 to 0.32 g g^−1^, when 12% and 23% (v/v) hydrolysate, respectively, was used in the propagation step.

As discussed above, the XR/XDH pathway has different co-factor preferences for the two enzymes – (NAD(P)H in the case of XR and NAD+ in the case of XDH – which leads to redox imbalance and xylitol production at the expense of ethanol production.

Advantageously, when cells were grown in 23% (v/v) hydrolysate, the xylitol yield was as low as 0.08 g g^−1^ despite the high amount of xylose consumed, as compared with 0.15 g g^−1^ when 12% (v/v) hydrolysate was used in the propagation. This could be due to an improved capacity for furfural detoxification acquired during propagation with hydrolysate; it is known that furfural can act as an external electron acceptor, re-oxidizing NADH and reducing xylitol secretion (Ask *et al*., 2013). However, further experiments would be necessary to understand this interesting effect.

These results clearly show that choosing a propagation strategy that leads to more robust and better-adapted cells leads to drastically improved fermentation performance.

### Quantitative polymerase chain reaction

For quantitative polymerase chain reaction (qPCR) analysis, a cell suspension (2 ml) from propagation cultures was centrifuged at 4000 r.p.m. for 3 min, washed with 0.9% NaCl and centrifuged again. The pellet was frozen in liquid nitrogen and stored at −80°C until RNA extraction.

The expression of genes related to tolerance to inhibitors and stress resistance, i.e. *ADH6*, *ALD6*, *CTA1* and *ZWF1*, was studied in cells propagated under different conditions.

The *TAF10* gene was used as internal reference gene. It was found to have stable expression in all samples because its Ct value did not vary significantly. The primer sequences used in the analysis were designed from the sequences listed in the Saccharomyces Genome Database (http://www.yeastgenome.org/) and stated in Table 1. Furthermore, the relative quantification of gene expression was evaluated using the comparative ΔΔCt method (Livak and Schmittgen, 2001).

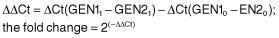


**Table 1:** Primer sequences used in the qPCR analysis

Gene	Forward primer	Reverse primer
*ADH6*	GTCTTGGTGGTATCGGCAGTATGGGTA	ATGTCGGTAAGGGAGGAAGCACAGACTA
*ALD6*	ACCCAAGAGAAAGAGGCCGTCTACTAAG	GCTCTAAGGTGGTGAAGTTCATGTAGCC
*CTA1*	CAGTACGGTAAATCTGAGGACGGGTCT	GACCGCTTTGTACTGCAGTCTGATCTC
*ZWF1*	GACATTACTGATATCTGCGGGTCTGCT	GGGAACTTGGAAGGGTCTCTGATAAAG
*TAF10*	TACCCGAATTTACAAGAAAAGATAAGA	ATTTCTGAGTAGCAAGTGCTAAAAGTC

**Figure 3:**
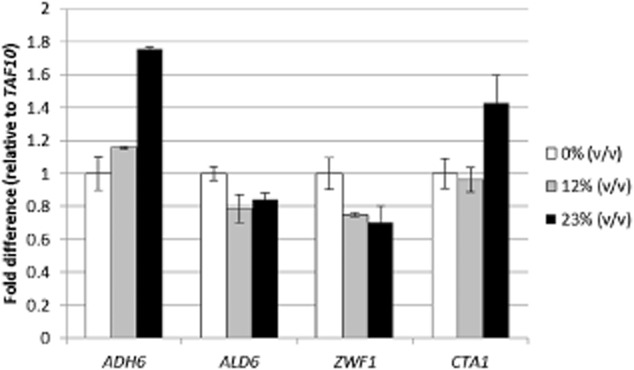
The RNA was extracted using an RNeasy kit (Qiagen) with DNase treatment, according to the manufacturer's protocol. The samples were subjected to reverse transcription and the cDNA was then used for qPCR. Expression of *ADH6*, *ALD6*, *ZWF1* and *CTA1* relative to *TAF10* in *S. cerevisiae*. Expression of these genes related to resistance to inhibitors and to the stress response was quantified using Brilliant® II SYBRGreen QPCR Master Mix, forward and reverse primers (0.5 μM each) and 2 μl cDNA. The qPCR procedure was performed using a Stratagene Mx3005P instrument. The qPCR program started with an initial denaturation for 10 min at 95°C. Amplification consisted of 40 cycles of 30 s at 95°C and 1 min at 65°C. This was followed by 1 min at 72°C for elongation of the amplicons.

*ADH6* encodes an alcohol dehydrogenase, and *ALD6* encodes an aldehyde dehydrogenase. Both enzymes are involved in resistance to phenolic fermentation inhibitors (Petersson *et al*., 2006; Park *et al*., 2011). It has even been shown that yeast clones that overexpress *ADH6* have increased capacities for reducing furfural and HMF (Almeida *et al*., 2009). As indicated in Fig. 3, the expression of *ADH6* was upregulated when the cells were grown in the presence of hydrolysate. There was a 1.2-fold and a 1.8-fold difference when they were propagated on 12% and 23% (v/v) hydrolysate respectively. According to the fermentation results, the upregulation of this gene could indicate an improvement in the ethanol production process in the presence of inhibitors.

When the cells were grown in the presence of wheat straw hydrolysate, no significant differences were observed in *ALD6* expression (Fig. 3). However, this gene has been found to be upregulated during xylose fermentation in mineral media in the presence of HMF and furfural (Ask *et al*., 2013).

*ZWF1* encodes a cytoplasmic glucose-6-phosphate dehydrogenase that catalyses the first step in the pentose phosphate pathway, and it is also associated with sensitivity to furfural and HMF. Disruption of *ZWF1* has been found to increase the ethanol yield and reduce the xylitol yield in a xylose-fermenting recombinant strain of *S. cerevisiae* (Jeppsson *et al*., 2002). These findings were attributed to an altered flux through the pentose phosphate pathway and may explain the highest xylose fermentation ability observed in cells propagated with 12% (v/v) and 23% (v/v) hydrolysate when *ZWF1* expression was downregulated (Fig. 3).

*CTA1* encodes a catalase, which is induced under oxidative stress (Kim *et al*., 2006). Furthermore, it has recently been shown to be linked to tolerance to furfural and HMF (Kim and Hahn, 2013). Although no differences in *CTA1* expression were detected in cells grown at 0% (v/v) or 12% (v/v) hydrolysate, this gene was clearly upregulated when cells were propagated in 23% (v/v) hydrolysate, which may indicate that addition of hydrolysate during propagation also triggers the oxidative stress response.

The qPCR results clearly show that the propagation strategy followed led to changes in stress associated genes. However, the relationship between the observed changes in gene expression and ethanol production will require further research to be fully understood.

## Conclusions

Hydrolysate addition during the propagation step pre-adapts the cells to the inhibitors, leading to cells with improved specific growth rates and reduced lag phases in minimal medium with vanillin and acetic acid. Furthermore, the propagation strategy also had a huge effect on the performance of the cells in ethanol production from wheat straw hydrolysates increasing the final ethanol production yields by 80%.

Cells propagated according to an appropriate strategy also show high xylose consumption rates, high ethanol production rates and low xylitol yields.

Depending on the propagation strategy, changes can be found in gene expression and thereby highlight the importance of optimizing the propagation step for achieving efficient ethanol production.
